# S-Equol enhances osteoblastic bone formation and prevents bone loss through OPG/RANKL *via* the PI3K/Akt pathway in streptozotocin-induced diabetic rats

**DOI:** 10.3389/fnut.2022.986192

**Published:** 2022-10-21

**Authors:** Zhe Xu, Jing Xu, Shuo Li, Hanqiang Cui, Guiming Zhang, Xiangmin Ni, Jian Wang

**Affiliations:** ^1^Department of Nutrition, Xinqiao Hospital, Army Medical University (Third Military Medical University), Chongqing, China; ^2^Department of Endocrinology, Xinqiao Hospital, Army Medical University (Third Military Medical University), Chongqing, China

**Keywords:** diabetic osteoporosis (DOP), S-Equol, estrogen receptor β (ERβ), AKT, osteoprotegerin (OPG), receptor activator of nuclear factor kappa-B ligand (RANKL)

## Abstract

**Background:**

This study aimed to explore whether S-Equol delays diabetes-induced osteoporosis and the molecular mechanisms underlying its therapeutic effects.

**Materials and methods:**

Thirty-five male Sprague–Dawley rats were randomized into five groups. The diabetic osteoporosis (DOP) group and three S-Equol treatment groups were intraperitoneally injected with streptozotocin (STZ) to develop a DOP model. After the 12-week intervention, bone transformation indicators were detected using an enzyme-linked immunosorbent assay kit; bone mineral density (BMD) and bone microstructure were obtained using dual-energy X-ray absorptiometry and microCT; morphological changes in the bone tissue were investigated using HE staining; bone morphogenetic proteins were detected using immunohistochemical staining. ROS17/2.8 cells were cultured *in vitro*, and Cell Counting Kit-8 was used to test the protective effects of S-Equol in osteoblastic cells in a high-fat and high-glucose environment. Furthermore, the expression of osteoprotegerin (OPG), receptor activator of nuclear factor kappa-B ligand (RANKL), estrogen receptor β(ERβ), phosphorylated Akt (pAKT)/protein kinase B (AKT), and osteocalcin (OC) in bone tissue and ROS17/2.8 cells was assessed using reverse transcription polymerase chain reaction (RT-PCR) and western blotting. To determine whether ERβ and phosphatidylinositol 3’ -kinase (PI3K)/AKT signaling pathways are involved in the process, LY294002 (PI3K signaling pathway inhibitor) and small interfering RNA targeting ERβ mRNA (si-ERβ) were used to verify the function of the ERβ-mediated PI3K/AKT pathway in this process.

**Results:**

After the 12-week intervention, S-Equol enhanced BMD, improved bone microarchitecture in DOP rats (*P* < 0.05), and improved markers of bone metabolism (*P* < 0.05). *In vitro*, 10^–6^ mmol/L S-Equol was selected to significantly protect osteoblasts from high- and high-glucose environments (*P* < 0.05). Gene expression of OPG, ERβ, pAKT/AKT, and OC was upregulated compared to the DOP group, and RANKL was downregulated compared to the DOP group (*P* < 0.05) both in bone tissue and osteoblastic cells. The promotion of OPG and pAKT/AKT is mediated by LY294002 and siERβ.

**Conclusion:**

S-Equol binds to ERβ to regulate OPG/RANKL *via* the PI3K/AKT pathway and improve DOP. Our results demonstrate the potential role of S-Equol in the treatment of DOP by targeting ERβ. Thus, S-Equol may have the potential to be an adjuvant drug for treating DOP.

## Introduction

The incidence of diabetes mellitus is increasing at an alarming rate, imposing tremendous suffering and economic burden on patients (1). Associated with reduced bone mineral density (BMD) and a deteriorated microarchitectural structure of bone tissue, osteoporosis causes a high risk of fractures. Osteoporosis entails large health and economic burden worldwide too (2). There is a known risk of premature mortality in patients (3). A recent meta-analysis based on 42 studies with a total of 17,571,738 cases found diabetes increases the risk of hip fracture (4). Studies have shown that the pathogenesis of diabetic osteoporosis (DOP) may be closely related to hyperglycemia, ferroptosis, reactive oxygen species (ROS), advanced glycation end products (AGEs) in collagen and inflammatory mediators (5–7). In addition, an altered pattern of adipocyte function is a critical mechanism responsible for DOP (8).

Osteoblastic cells secrete a large amount of osteoprotegerin (OPG) and receptor activator of nuclear factor kappa-B ligand (RANKL). RANKL facilitates the differentiation of osteoclast precursors into osteoclasts, resulting in bone resorption. OPG functions as a decoy receptor for RANKL and inhibits RANKL-induced osteoclast differentiation. Previous studies have indicated that the balance between OPG and RANKL is disrupted in a diabetic environment, and as a result, the equilibrium between bone formation and bone absorption is disrupted (9). IGF-1 activates mTOR through the PI3K-Akt pathway to induce the differentiation of MSCs into osteoblasts (10) and inhibits osteoblast apoptosis, thereby promoting osteoblastic growth (11). Indeed, there have been some scientific evidences that the PI3K/Akt signaling pathway is related to the OPG/RANKL ratio (12, 13). Based on our experience and a close reading of the literature, the PI3K-AKT signaling pathway can regulate the balance between RANKL and OPG (OPG/RANKL ratio) secreted by osteoblasts.

Equol [7-hydroxy-3-(4’-hydroxyphenyl)-chroman] is a metabolite of the isoflavone daidzein in the gut of humans and animals by certain gut bacteria, which has a high affinity for estrogen receptors (14, 15). Many kinds of animal species such as mice, rats, sheep, goats and chickens have been confirmed to produce Equol after soy or daidzein consumption. The focus on S-Equol results from the equol hypothesis (16) that equol producers can benefit from consuming S-Equol. S-Equol has a relatively higher affinity for estrogen receptor β (ERβ) (16, 17) which is the predominant ER in vascular endothelium, bone, and male prostate tissues (18) compared with the isoflavone daidzein. Currently, numerous studies indicate that S-Equol has antioxidant activity, improves menopause-associated symptoms, and reduces the incidence of breast cancer (19). In particular, S-Equol protects against osteoporosis, and osteoporotic fractures occur frequently in postmenopausal women owing to decreased estrogen levels (20, 21). However, no more than 60% of people can produce Equol from daidzein in their daily diet.

Studies have revealed that daidzein can increase the OPG/RANKL ratio in MG-63 osteoblast cells (22) and genistein can protect against ovariectomy-induced bone loss by upregulating the OPG/RANKL ratio (23). Concurrently, there are data that S-Equol, through PI3K/Akt activation, can protect chondrocytes against osteoarthritis, leading to apoptosis (24). However, the role of S-Equol in diabetic osteoporosis (DOP) and whether S-Equol regulates the balance between OPG and RANKL remain unclear. Our study was designed to determine the role of S-Equol in *in vitro* and *in vivo* DOP models, determine whether S-Equol regulates the balance between OPG and RANKL *via* the PI3K-AKT pathway, and identify potential therapeutic targets for clinical treatment.

## Materials and methods

### Animal experimental protocols

All animal protocols were approved by the Experimental Animal Ethics Committee of the Third Military Medical University (AMUWEC2019350, 12 March 2019). Thirty-five male Sprague–Dawley rats aged 6 weeks (Huafukang Co., Ltd., Beijing, China) were housed under controlled room temperature (RT) (22 ± 2°C) and humidity (50–70%) with a 12 h/12 h day/night cycle with free access to water and food. Rats, except for those in the control group, were fed a high-sugar and high-fat diet containing 10% lard, 20% sucrose, 2% cholesterol, 0.5% sodium cholate, and 67.5% normal diet during the former 4-week experiment period. After 4 weeks of high-sugar and high-fat feeding, according to the safety dose from reference (25, 26), the rats were randomly divided into five groups: CON (control), DOP (diabetic osteoporosis), DOP + S-Equol (low-dose [LD], 20 mg/kg), DOP + S-Equol (medium-dose [MD], 40 mg/kg), and DOP + S-Equol (high-dose [HD],80 mg/kg). Rats in the DOP group, DOP + S-Equol (20 mg/kg), DOP + S-Equol (40 mg/kg), and DOP + S-Equol (80 mg/kg) were intraperitoneally injected with 25 mg/kg STZ (Beyotime) dissolved in 0.1 mM vehicle citrate buffer (pH 4.5) to induce diabetes. The rats in the control group were injected with vehicle citrate buffer (2 mL/kg). These rats were considered diabetic, whose fasting blood glucose levels from the tail vein were over 16.7 mM, 7 days after STZ injection. Blood glucose levels were assessed using a portable glucometer with compatible glucose test strips (OneTouch^®^Ultra^®^, LifeScan, Milpitas, CA, USA). One week later, the diabetic rats in the DOP + S-Equol (20 mg/kg, *n* = 7), DOP + S-Equol (20 mg/kg, *n* = 7), and DOP + S-Equol (80 mg/kg, *n* = 7) groups were gavaged with S-Equol [100152, Daicel Chiral Technologies (China) Co., Ltd., Shanghai, China] dissolved in 1% DMSO + 99% corn oil at a dose of 20, 40, and 80 mg/kg daily, respectively. The rats in the CON and DOP groups were gavaged with the same volume of 1% DMSO + 99% corn oil. Fasting blood glucose levels in the tail vein and body weight were measured once weekly. The rats were sacrificed 16 weeks after S-Equol treatment. The serum was separated by centrifugation and stored at −80°C until analysis. Meanwhile, bilateral femora were isolated, collected, and stored at −80°C until analysis.

### Bone mineral density and microCT

Bone mineral density was measured under general anesthesia. The anesthetized rats were positioned in ventral recumbency on the scan table. All scans were performed using dual-energy X-ray absorptiometry (DXA) to evaluate the BMD in rats at week 16. All DXA scans were analyzed using the manufacturer’s recommended software (Encore 2011; GE Healthcare). The right femur metaphysis was used for structural analysis of the bone-implant interface (*n* = 3). Bone tissue was subjected to micro-CT analysis (SkyScan1272; Bruker Micro-CT, Germany). Scanning was set to have the entire sample within the field of view throughout a full 360° rotation. The reconstruction was conducted using CTvox software (Data Viewer Version 1.6.0.0). Tissue volume, bone volume, percent bone volume, tissue surface, bone surface, intersection surface, bone surface/volume ratio, bone surface density, trabecular thickness, trabecular separation, trabecular number, and trabecular pattern factor were determined according to guidelines (CT-Analyzer Version 1.20.8).

### Biochemical assay for bone markers

OPG, OC, procollagen I N-terminal propeptide (PINP), 25(OH)D3, receptor activator for RANKL, type 1 collagen N-terminal breakdown products (NTX), and pyridinoline (PYD) of serum were measured using an enzyme-linked immunosorbent assay (ELISA) kit (Xiamen Huijia Biotechnology Co., Ltd., Xiamen, China). ELISA was performed in accordance with the manufacturer’s instructions.

### Histopathological analysis, oil red O staining and immunohistochemical analysis of receptor activator of nuclear factor kappa-B ligand, osteoprotegerin, estrogen receptor β, and estrogen receptor α

Five femoral diaphyses per group were harvested, fixed in 4% paraformaldehyde for 48 h, and decalcified with 10% EDTA (pH 7.5) for 15 days. Following decalcification, specimens were dehydrated and embedded in paraffin; 4-μm thick sections were obtained in the coronal plane. Histomorphometric evaluations were performed and evaluated using light microscopy (40X magnification). Thin sections of the femoral diaphysis (4 μm) were obtained using a microtome and transferred to gelatin-coated slides. Frozen sections were counterstained with hematoxylin solution and oil red O solution to visualize the buildup of lipid droplets in the bone marrow. The oil red O-positive regions were measured using ImageJ software after each specimen was inspected under light microscopy (40X magnification). After deparaffinised and rehydrated the femoral diaphysis tissue slices were washed with 0.3% Triton X-100 in phosphate buffer, quenched with endogenous peroxidase (3% hydrogen peroxide), and incubated with the following primary antibodies overnight at 4°C: RANKL, 1:600; OPG, 1:500; ERβ,1:400; and estrogen receptor α (ERα), 1:400. After primary antibody incubation, the sections were washed with phosphate buffer and incubated with streptavidin-HRP-conjugated secondary antibody for 30 min. The sections were scanned using CaseViewer software, and measurements were performed using ImageJ software.

### Cell cultures

ROS17/2.8 cells, a rat osteoblastic cell line, were obtained from Procell Life Science and Technology Co., Ltd., ROS17/2.8 cells were cultured in DMEM low-glucose culture medium (Procell Life Science and Technology Co., Ltd., China) supplemented with 10% FBS and 1% penicillin/streptomycin. The cells were cultured in a humidified atmosphere of 95% air and 5% CO2 at 37°C and subcultured every 3 days. ROS17/2.8 cells were cultured in type 2 diabetes mellitus (T2DM)-mimic conditions in osteogenic DMEM high-glucose culture medium and 150 mmol/L sodium palmitate. ROS17/2.8 cells were incubated in regular culture medium containing different concentrations of S-Equol (10^–9^–10^–5^mmol/L) for 48 h after exposure to T2DM-mimic conditions for 24 h. Cell Counting Kit-8 (CCK8) colorimetric assays were performed to detect cell viability.

### Small interfering RNA transfection and PI3K inhibition

For transfection, 1 × 10^5^ cells per well in a 6-well dish were seeded in DMEM high-glucose culture medium containing 15% FBS. siRNA against ERβ mRNA was obtained from Gene Pharma (Gene Pharma Co., Ltd., China). We designed three siRNA sequences for ERβ (respectively named ER β-1763, ER β-619, and ER β-537), one negative control sequence, one positive control (β-actin), and one negative control (FAM). The siRNA sequences are listed in Table 1. The transfection protocol is as follows: there were two centrifuge tubes, one contained 150 pmol si-NC, Si-Actin, or si-ERβ with serum-free DMEM high-glucose culture medium and the other contained 8-μL GP-trasfect-Mate with DMEM high glucose culture medium, which was then reacted for 5 min. The mixture, which included two kinds of ingredients, was mixed well and incubated for 15 min at RT, and then the mixture was added to the plate and was changed after 8 h; 48 h later, transfection efficiency was verified using quantitative RT-PCR. For PI3K inhibition, ROS17/2.8 cells were treated with the PI3K inhibitor LY294002 (25 μmol/L, Beyotime, Shanghai, China) in a 6-well dish for 30 min. after 48 h S-Equol intervention, Western blotting and RT-PCR were performed.

**TABLE 1:** Sequences of the small interfering RNA (siRNA) involved in this study.

Names	Sequence(5′-3′)
Rat-Negative control–Sence	UUCUCCGAACGUGUCACGUTT
Rat-Negative control–Antisence	ACGUGACACGUUCGGAGAATT
Rat-Positive control–Sence	CUCUGAACCCUAAGGCCAATT
Rat-Positive control–Antisence	UUGGCCUUAGGGUUCAGAGGG
Rat-ER β-1763–Sence	GCUGAAUGCUCACACGCUUTT
Rat-ERβ-1763–Antisence	AAGCGUGUGAGCAUUCAGCTT
Rat-ERβ-619–Sence	GAGCACACCUUACCUGUAATT
Rat-ERβ-619–Antisence	UUACAGGUAAGGUGUGCUCTT
Rat-ERβ-537–Sence	AUGUCAUAGCUGAAUACUCAU
Rat-ERβ-537–Antisence	GAGUAUUCAGCUAUGACAUUC

### Western blot

Western blotting analysis of total proteins from the femoral metaphysis or cells was performed. The tissue of the femoral metaphysis suspension was further ground with 4–5 mm steel grinding balls at 60 cycles/s for 5 min in RIPA lysis buffer (RIPA, Beyotime, Shanghai, China) with 1% phenylmethylsulfonyl fluoride (PMSF) in a tissue homogenizer (Qiagen TissueLyser LT, Germany) at 4°C. Cells were lysed with RIPA buffer supplemented with 1% PMSF and lysed by sonication on ice for 10 min. The insoluble materials were removed by centrifugation at 12,000 × *g* for 10 min, and the supernatants were collected. The amount of protein was quantified using the BCA protein assay (Sangon, Shanghai, China). The protein sample was denatured in boiling water for 5 min in SDS-PAGE sample loading buffer (Sangon, Shanghai, China). Aliquots of the samples (femoral metaphysis 40 μg, cells 20 μg) were then subjected to SDS-PAGE on 10% gels under reducing conditions and electroblotted onto PVDF membranes (IPVH00010, 0.45 μm, Millipore, USA). The membranes were blocked with 5% fat-free dry milk in TBST (0.1% Tween-20 and 0.1 M NaCl in 0.1 M Tris–HCl, pH 7.5) for 1 h at room temperature (RT) and then incubated with primary antibodies and placed in an overhead shaker at 4°C for 8 h. The antibodies used were anti-OPG (33i3914, Affinity, Jiangsu, China), anti-RANKL (23408-1-AP, Proteintech), anti-ERβ (14007-1-AP, Proteintech), anti-AKT (10176-2-AP, Proteintech), anti-pAKT (S473, Beyotime), anti-β-actin (ab8226, Abcam, Shanghai, China), and anti-glyceraldehyde-3-phosphate dehydrogenase (GAPDH) (ab9485, Abcam). The membranes were then incubated with a horseradish peroxidase-conjugated secondary antibody (1:5000, Beyotime) at RT for 1 h, followed by chemiluminescence detection (P0018, Beyotime). Each incubation step was followed by three washes (10 min each) with TBST. Protein bands were quantitatively analyzed using an image analysis system (Quantity One software; BioRad ChemiDoc, BioRad, USA).

### qRT-PCR

According to the instructions, total RNA was prepared using TRIzol reagent (TAKARA BIO INC., Beijing, China). Briefly, 100 mg of femoral metaphysis tissue or cells from each well of six-well plates were homogenized in 1 mL of TRIzol reagent. After 5 min stain at RT and centrifugation, the RNA was extracted with chloroform and precipitated with isopropyl alcohol. RNA samples were then re-suspended in 50 μL of DEPC-treated H2O; 5 μg of total RNA was used in the first-strand cDNA Synthesis Kit (Beyotime). GAPDH was used as an internal control. The quantitative RT-PCR primers are listed in Table 2. The PCR mixture (10 μL final volume per reaction) was prepared in accordance with the manufacturer’s protocol. Amplifications were performed under the following conditions: 95°C for 5 min, then 45 cycles at 95°C for 5 s, 59°C for 15 s, 72°C for 20 s, and 65°C for 5 min on a PCR System. All RT-PCR reactions were performed using the Bio-Rad CFX Connect system.

**TABLE 2:** Primer sequences of the genes involved in this study.

Primer	Sequence(5′-3′)
Rat-β-actin-F	CTCTGTGTGGATTGGTGGCT
Rat-β-actin-R	CGCAGCTCAGTAACAGTCCG
Rat-OC-F	TTATTGTTTGAGGGGCCTGGG
Rat-OC-R	TGCTCCTACAAAGCTGTCTCC
Rat-OPG-F	TGAGACGTCATCGAAAGCAC
Rat-OPG-R	CGCACAGGGTGACATCTATT
Rat-ERβ-F	CGTCAGGCACATCAGTAACAAG
Rat-ERβ-R	GGACAATCCTTCCAAATCAGAC
Rat-RANKL-F	TTCAGAATTGCCCGACCAGTTTTT
Rat-RANKL-R	CCCAGACATTTGCACACCTCAC

### Statistical analysis

Data are presented as the mean ± standard deviation (SD). Analyses were conducted using R (R statistics^1^), version 4.2.0. The normality of the parameters was assessed using Shapiro test, the homogeneity of the parameters was assessed using Levene’s test, and parameter distribution and pairwise comparisons among groups were analyzed *via* ggbetweenstats of Package ggstatsplot (version 0.9.3) (27). All data were analyzed using one-way ANOVA followed by the LSD *post hoc* test. *P* < 0.05 was considered statistically significant.

## Results

### Effects of S-Equol on body weight, fasting blood glucose, serum albumin-corrected calcium, and phosphorus levels in diabetic osteoporosis rats

**FIGURE 1:**
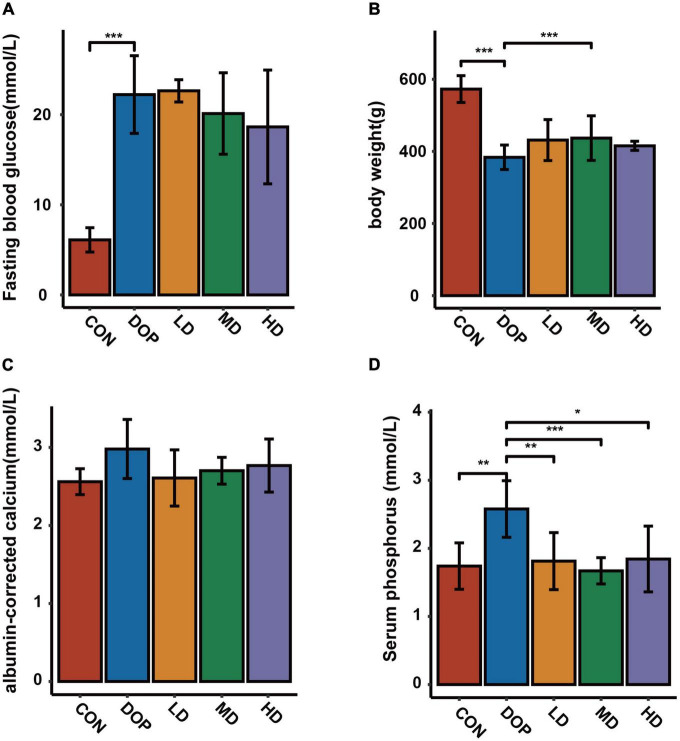
Effects of S-Equol on body weight, fasting blood glucose, serum albumin-corrected calcium and phosphorus levels in different groups of rats after 12 weeks of gastric gavage. **(A)** Fasting blood glucose levels; **(B)** body weight of rats; **(C)** serum albumin-corrected calcium levels; **(D)** serum phosphorus levels. All data are expressed as mean ± SD (*n* = 7 for body weight and fasting blood glucose levels). *p < 0.05, **p < 0.01, ***p < 0.001 as compared to the DOP group.

The body weights and fasting blood glucose levels of the rats at the end of the experiment are shown in Figures 1A,B. Compared with the rats in the CON group, the rats in the DOP groups displayed a higher level of fasting blood glucose (*P* < 0.05). S-Equol treatment did not have a significant effect on fasting blood glucose levels in diabetic rats. The body weight in the DOP groups was significantly lower than that in the CON group (*P* < 0.05), and medium-dose S-Equol treatment caused a significant increase in the body weight of diabetic rats (*P* < 0.05). The serum albumin-corrected calcium and phosphorus levels in DOP rats are shown in Figures 1C,D. Serum phosphorus levels were significantly increased in DOP rats (*P* < 0.05), while there was no trend in the serum levels of albumin-corrected calcium. S-Equol decreased serum phosphorus but had no significant effect on serum albumin-corrected calcium levels in DOP rats (*P* < 0.05).

### S-Equol enhances bone mineral density in diabetic osteoporosis rats

**FIGURE 2:**
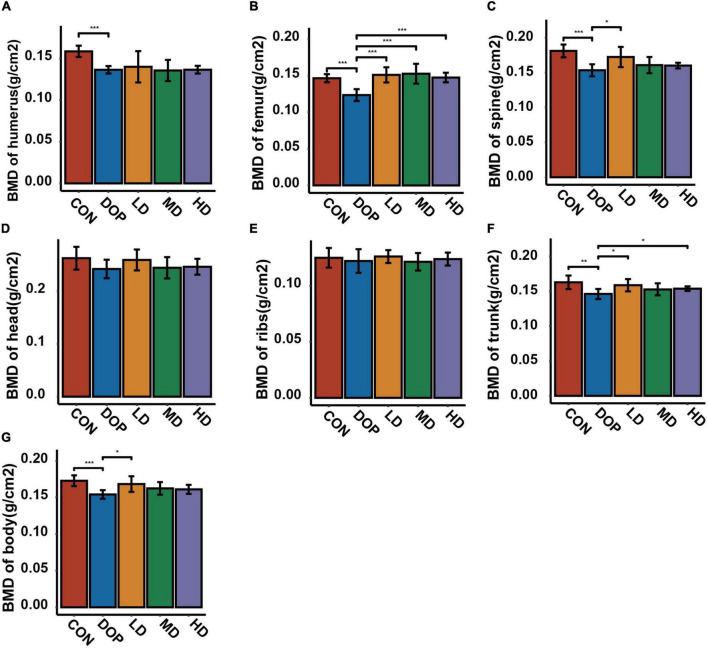
Effects of S-Equol on bone mineral density (BMD) in different groups of rats after 12 weeks of gastric gavage. **(A)** BMD of the humerus; **(B)** BMD of the femurs; **(C)** BMD of the spine; **(D)** BMD of the head; **(E)** BMD of the ribs; **(F)** BMD of the trunk; **(G)** BMD of the body. All data are expressed as mean ± SD (*n* = 5 for various parts of BMD). *p < 0.05, **p < 0.01, ***p < 0.001 as compared to the diabetic osteoporosis (DOP) group.

Bone mineral density (BMD) analyses were used to evaluate the changes in various parts of the skeleton in DOP rats. As shown in Figure 2, the results revealed an obvious decrease in BMD of the humerus, femur, trunk, and body in DOP rats compared with CON rats (*P* < 0.05). S-Equol treatment could improve the BMD of the above parts, while there was a significant effect on the BMD of the femurs (*P* < 0.05).

### S-Equol improves the bone microarchitecture in diabetic osteoporosis rats

**FIGURE 3:**
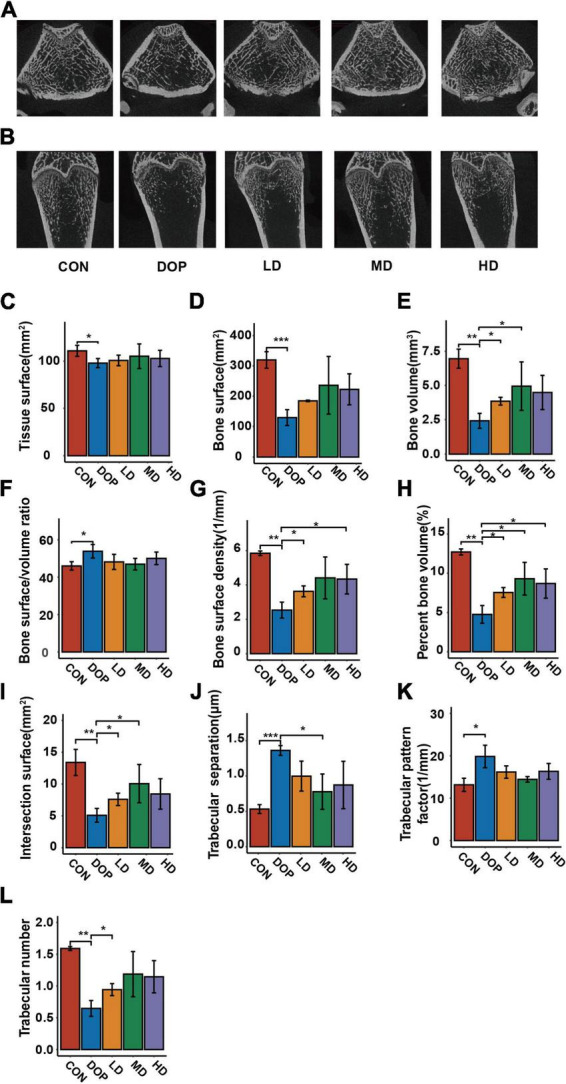
Effects of S-Equol on histological morphometric alteration of the femoral metaphysis in different groups of rats. **(A)** Representative microcomputed tomography coronal section images of femoral trabecular bone micro-architecture; **(B)** representative microcomputed tomography coronal section images of femoral trabecular bone micro-architecture; **(C)** tissue surface; **(D)** bone surface; **(E)** bone volume; **(F)** bone surface/volume ratio; **(G)** bone surface density; **(H)** percent bone volume; **(I)** intersection surface; **(J)** trabecular number; **(K)** trabecular separation ratio; **(L)** intersection surface. All data are expressed as mean ± SD (*n* = 3). *p < 0.05, **p < 0.01, ***p < 0.001 as compared to the diabetic osteoporosis (DOP) group.

MicroCT has become an important tool for the analysis of bone morphology and has been used to evaluate alterations in the trabecular bone microarchitecture in diabetic rats. As shown in Figures 3A,B, micro-CT images of the sagittal and coronal planes of the femurs showed an obvious decrease in trabecular bone mass and deterioration of the cancellous bone microarchitecture in the DOP group compared with the CON group. S-Equol treatment for 12 weeks showed a significant protective effect against trabecular bone architectural deterioration in rats with DOP. As shown in Figures 3C–L, the bone histological morphometric analysis revealed that tissue surface, bone surface, bone volume, bone surface density, percent bone volume, intersection surface, and trabecular number were significantly reduced (*P* < 0.05), and bone surface/volume ratio, trabecular separation ratio, and trabecular pattern factor were significantly increased in the femurs of DOP rats (*P* < 0.05). S-Equol treatment improved the above parameters but only exerted significant effects on bone volume, bone surface density, percent bone volume, intersection surface, trabecular number, and trabecular separation ratio in the femurs of DOP rats.

### S-Equol shows histological improvement of the proximal part of the femoral metaphysis

**FIGURE 4:**
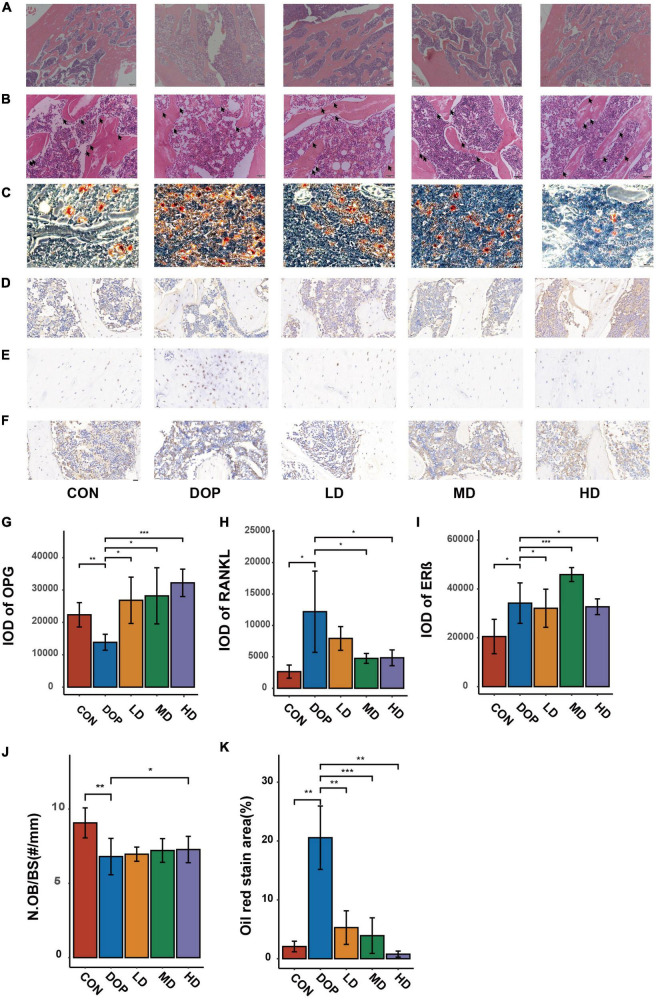
Effects of S-Equol on hematoxylin and eosin staining histological changes, oil red O stain and immunohistochemical analyses of the distal part of the femoral metaphysis in different groups of rats. **(A)** Objective lens with a magnifying power of × 4 (Scale bars: 200 μm); **(B)** Objective lens with a magnifying power of × 20 (Scale bars: 50 μm); **(C)** Oil red O stain analysis of paraffin sections of the ictal part of the femoral metaphysis Objective lens with a magnifying power of × 4 (Scale bars: 200 μm); Paraffin sections of the ictal part of the femoral metaphysis were prepared for immunohistochemical staining of OPG **(D),** RANKL **(E)**, and ERβ **(F)**. The expression of biomarkers was scanned and evaluated by semiquantitative analysis with CaseViewer and ImageJ software **(G–I)**. Number of osteoblasts per trabecular surface of bone (N.Ob/BS) **(J)**. Quantitative analysis of Oil red O stain analysis **(K)**. All data are expressed as the mean ± SD (*n* = 5). *p < 0.05, **p < 0.01, ***p < 0.001 as compared to the diabetic osteoporosis (DOP) group.

As shown in Figures 4A–C,J,K, compared with the CON group, the trabecular bones in the femurs of DOP rats became thinner and irregular, the trabecular bone reticulate structure was disorganized and large amount of marrow fat content in bone marrow cavity under low magnification. Under high magnification, large numbers of fat-forming vacuoles were observed in the bone marrow cavity and less number of osteoblasts per trabecular surface of bone in DOP rats (*P* < 0.05). After S-Equol intervention, in rats of the S-Equol-treated groups, there were obvious improvements in trabecular number, trabecular space, number of osteoblasts per trabecular surface of bone (*P* < 0.05), and trabecular thickness. In addition, a significant decrease in volume and number of fat-forming vacuoles were observed after treatment (*P* < 0.05).

### Immunohistochemical analysis on markers of bone loss and estrogen receptor β

As shown in Figures 4D–I, immunohistochemical analyses demonstrated that the expression of RANKL and ERβ in DOP rats was significantly increased, while the expression of OPG in DOP rats was significantly increased compared to that in the CON group. Compared to the DOP group, the expression of RANKL was decreased in the S-Equol treatment groups. OPG and ERβ expression in the S-Equol treatment group was higher than that in the DOP group.

### The changes in bone metabolism in serum

**FIGURE 5:**
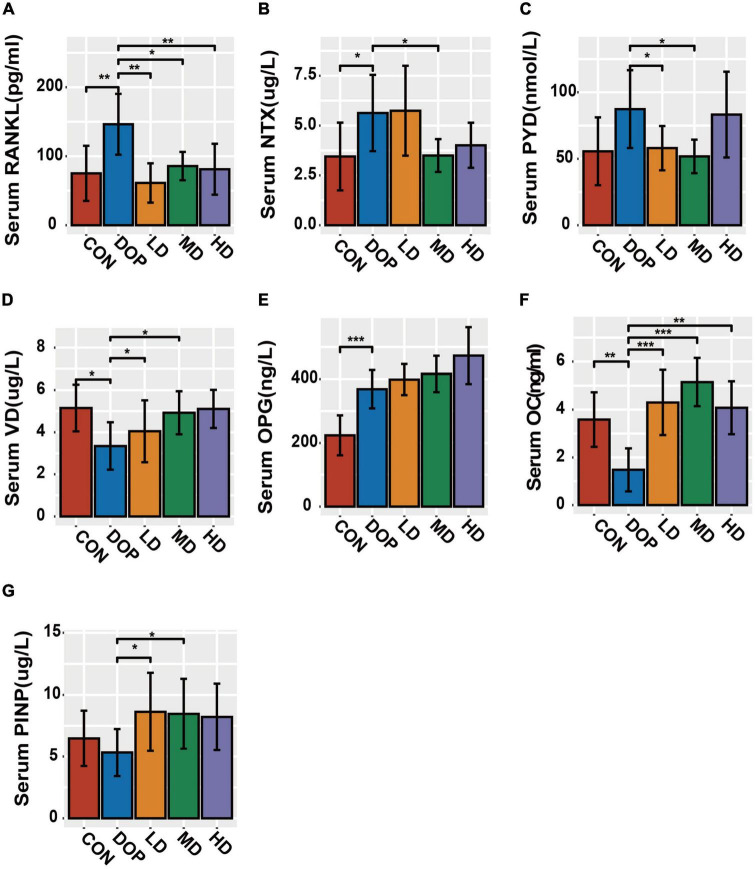
Bone metabolism in serum biomarker analysis in different groups of rats. **(A)** Serum RANKL levels; **(B)** serum NTX levels; **(C)** serum PYD levels; **(D)** serum VD levels; **(E)** serum OPG levels; **(F)** serum OC levels; **(G)** serum PINP levels. All data are expressed as the mean ± SD (*n* = 5). *p < 0.05, **p < 0.01, ***p < 0.001 as compared to the diabetic osteoporosis (DOP) group.

As shown in Figure 5, compared to the CON group, the levels of NTX, RANKL, and OPG were significantly increased in the DOP group (*P* < 0.05). The DOP model induction caused a decrease in serum 25(OH)D3 and OC expression (*P* < 0.05). S-Equol treatment significantly decreased sRANKL, NTX, and PYD levels and increased VD, OC, and PINP levels in the serum compared to those in the DOP group (*P* < 0.05). S-Equol increased serum OPG but had no significant effect compared with DOP rats.

### S-Equol modulates the osteoprotegerin, receptor activator of nuclear factor kappa-B ligand, protein kinase B, and estrogen receptor β in the distal part of the femoral metaphysis tissues

**FIGURE 6:**
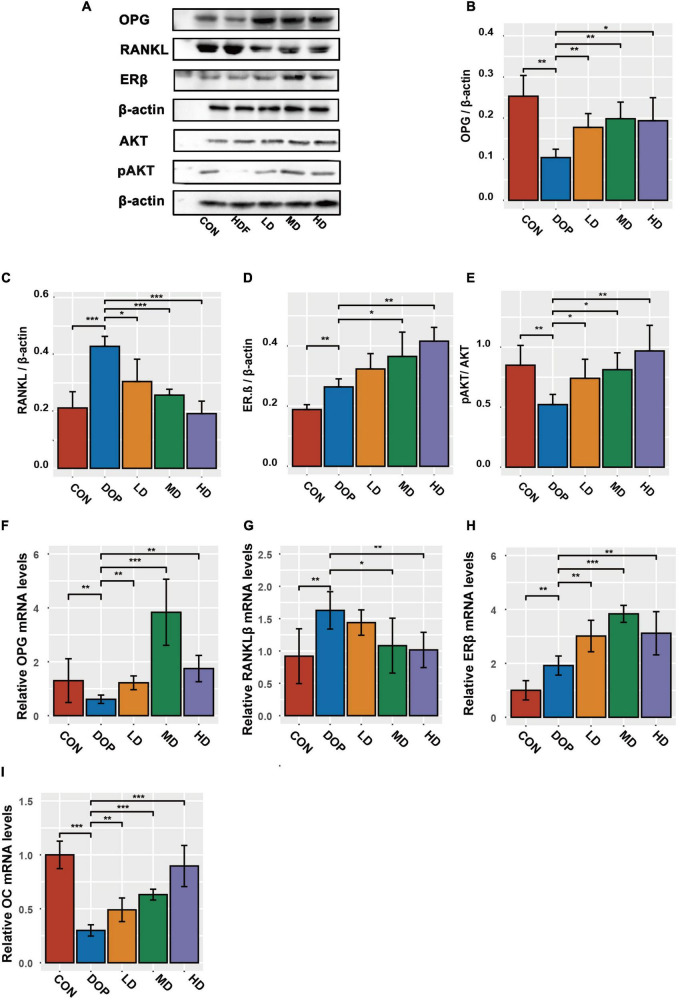
S-Equol is involved in the regulation of phosphorylation, osteoprotegerin/receptor activator of nuclear factor kappa-B ligand (OPG/RANKL) ratio, and level of estrogen receptor β (ERβ) in the distal part of the femoral metaphysis tissues of diabetic osteoporosis (DOP) rats. **(A–E)** The expression of key proteins was analyzed using the western-blot method; **(F–I)** the mRNA amounts of OC, OPG, RANKL, and ERβ were analyzed by the RT-PCR method. All data are expressed as the mean ± SD (*n* = 5). *p < 0.05, **p < 0.01, ***p < 0.001 as compared to the DOP group.

Phytoestrogens or S-Equol activated the PI3K-AKT pathway or regulated OPG/RANKL in the DOP rat model, which has been extensively reported to be involved in the improvement of osteoporosis; therefore, we examined whether the protective effect of S-Equol was associated with the AKT and OPG/RANKL pathways. We detected the protein levels of OPG, RANKL, ERβ, and pAKT/AKT in DOP rats after treatment with S-Equol using western blotting (Figures 6A–F). The protein levels of RANKL, pAKT/AKT ratio, and ERβ were markedly upregulated and OPG was markedly downregulated in DOP rats compared to those in the CON group (*P* < 0.05). After treatment with S-Equol, a marked upregulation of AKT phosphorylation, OPG/RANKL ratio, and level of ERβ was observed (*P* < 0.05). Consistent with the western blotting results, the mRNA levels of OC, OPG/RANKL ratio, and ERβ were conspicuously upregulated in comparison with the CON group (Figures 6F–I) (*P* < 0.05). However, the HD group (80 mg/kg S-Equol) showed reduced mRNA transcription of the OPG.

### S-Equol changes the expression of key regulator molecules of osteoblastic cells damaged by high-sugar and high-fat environment

**FIGURE 7:**
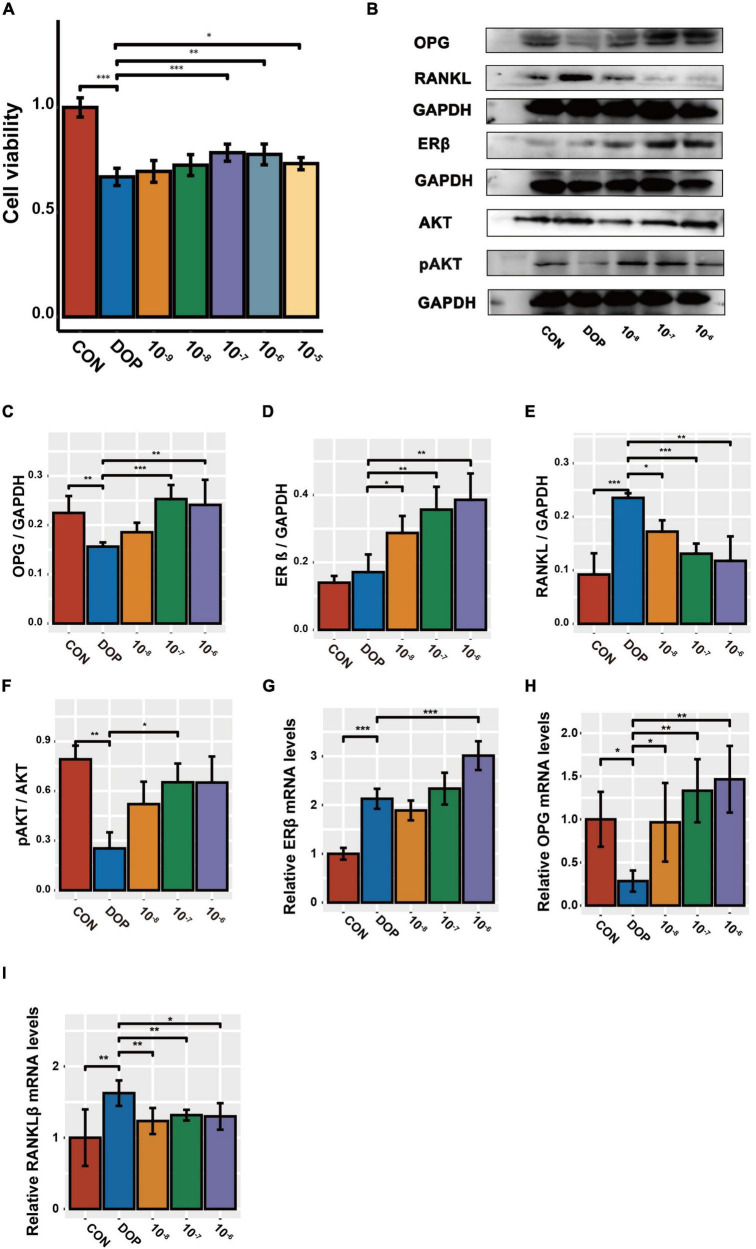
The effect of S-Equol on Pi3K/AKT signaling pathway and OPG/RANKL ratio in ROS17/2.8 cells. **(A)** After 48 h of S-Equol intervention, cell viability was detected by CCK8 assay; **(B–F)** The expression of OPG, RANKL, ERβ, and pAKT/AKT was analyzed using the western-blot method. **(G–I)** The amount of mRNA of OPG, RANKL, and ERβ was analyzed using the RT-PCR method. All data are expressed as the mean ± SD (*n* = 5). *p < 0.05, **p < 0.01, ***p < 0.001 as compared to the diabetic osteoporosis (DOP) group.

First, we treated ROS17/2.8 cells with different concentrations of S-Equol (10^–9^–10^–5^ mmol/L) in high-sugar and high-fat environments. It was found that cell viability by CCK-8 was significantly increased at 10^–7^ mmol/L while it was inhibited starting at a concentration of 10^–5^ mmol/L (Figure 7A. Therefore, we chose 10^–8^–10^–6^ mmol S-Equol for subsequent experiments. Similarly, in the *in vitro* experiments, the protein levels of ERβ, RANKL, and the pAKT/AKT ratio were markedly upregulated, and OPG was markedly downregulated in the high-sugar and high-fat environment (DOP) compared to the CON group. After treatment with S-Equol, a significant upregulation of AKT phosphorylation, OPG/RANKL ratio, and ERβ was observed (Figures 7B–F). Consistent with the western blotting results, the mRNA levels of the OPG/RANKL ratio and ERβ were markedly upregulated in comparison with the CON group (Figures 7G–I).

### The estrogen receptor β and PI3K-AKT signaling pathway plays a role in S-Equol-mediated amelioration of bone metabolism

**FIGURE 8:**
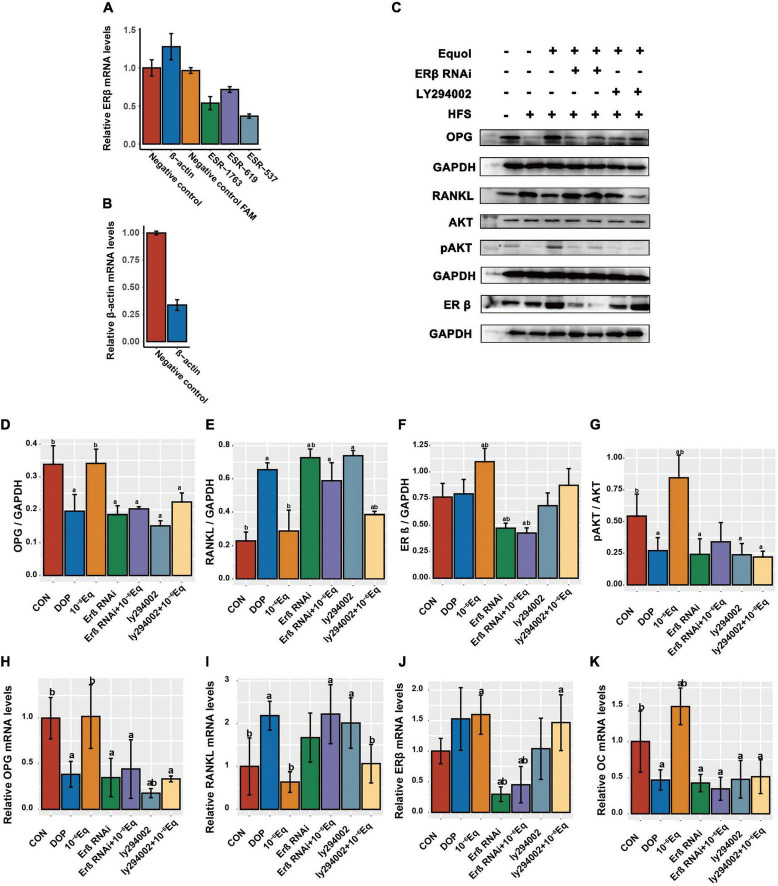
S-Equol intervention elevated the osteoprotegerin/receptor activator of nuclear factor kappa-B ligand (OPG/RANKL) ratio in ROS17/2.8 cells *via* the ERβ-PI3K-AKT pathway. **(A)** Quantitative RT-PCR was used to detect ERβ expression in ROS17/2.8 after siRNA transfection; **(B)** transfection with β-actin siRNAs was used as a positive control. **(C–G)** The protein expression level of osteoprotegerin (OPG), receptor activator of nuclear factor kappa-B ligand (RANKL), estrogen receptor β (ERβ), and phosphorylated Akt (pAKT/AKT) in treated ROS17/2.8 cells were assessed using western blotting. **(H–K)** The amounts of mRNA of OPG, RANKL, ERβ, and OC were measured using quantitative RT-PCR.

Knockdown of ERβ mRNA was confirmed using quantitative RT-PCR. Based on the *in vitro* and *in vivo* findings, we observed molecular alterations in the PI3K/AKT pathway and OPG/RANKL, and selected ESR-537 for transfection to further detect the expression of ERβ, OPG, RANKL, OC, and pAKT/AKT to explore the regulatory mechanisms of S-Equol in ROS17/2.8 cells (Figures 8A,B). As is shown in Figures 8C–K after 48 h of S-Equol treatment, the expression of RANKL, OC, OPG, p-AKT/AKT, and ERβ was not significantly different in ROS17/2.8 cells between the ERβ siRNA and ERβ siRNA + S-Equol groups (*P* > 0.05). This indicates that S-Equol regulates the expression of RANKL, OC, OPG, and p-AKT/AKT *via* ERβ and improves the expression level of ERβ to amplify this effect. Meanwhile, ROS17/2.8 cells treated with both LY294002 and S-Equol did not exhibit significantly higher or lower expression of OPG, OC, and pAKT than cells treated with LY294002 (*P* > 0.05); however, S-Equol increased the expression level of ERβ and decreased the expression level of RANKL (*P* < 0.05). Taken together, these results imply that activation of the PI3K-AKT signaling pathway *via* estrogen receptor β (ERβ) plays a protective role in S-Equol against DOP.

## Discussion

In the present study, we constructed a model of osteoporosis induced by T2DM. Decreased BMD, bone volume, bone surface density, trabecular number, and bone formation biomarkers and increased bone resorption biomarkers and trabecular separation were observed in DOP rats, indicating the validity of the animal model. After treatment with S-Equol for 12 weeks, the BMD, serum bone turnover markers, and bone structure of DOP rats recovered, indicating that S-Equol was able to ameliorate the severe bone loss induced by DOP. After treatment with S-Equol for 12 weeks, the BMD, serum bone turnover markers, and bone structure of DOP rats were significantly improved, indicating that S-Equol was able to ameliorate the severe bone loss induced by T2DM. Five main findings were derived from this study: (1) S-Equol prevents decreases in BMD in DOP rats, although there is no significant reduction of glycemia; (2) S-Equol increased bone mass and improved bone microstructure in DOP rats, however, S-Equol is not capable of wounded trabecular repair; (3) Treatment with S-Equol could affect the serum levels of OPG and RANKL, increasing the OPG/RANKL ratio and bone formation marker levels and decreasing bone turnover marker levels; (4) Treatment with S-Equol could significantly elevate the OPG/RANKL ratio, ERβ level, and p-Akt/Akt ratio in tibial metaphysis and ROS17/2.8 cells; and (5) S-Equol elevation of the OPG/RANKL ratio is due to ERβ receptor action *via* the PI3K/AKT pathway.

There is clear evidence that the risk of osteoporosis increases with the development of type 2 diabetes (28). This has become a global health issue that has attracted significant attention from the medical community. Many studies have investigated the pathogenesis of osteoporosis leading to T2DM. Increased advanced glycation end product (AGE) accumulation, which directly inhibits osteoblastic function, alters collagen cross-linking, and promotes an inflammatory environment in the bone and injury to the bone microstructure, is one of the leading causes of DOP (29). Insulin resistance and vitamin D deficiency that lead to hyperactivity of the parathyroid gland, which initiates calcium-phosphate metabolic dysregulation, are well-established causative factors for osteoporosis and bone loss (30–32). Although it is well-accepted that T2DM could increase the risk of osteoporosis and fragility fractures, several studies have indicated that T2DM is associated with an increase in BMD (33, 34), which is in contrast to our conclusion. Similarly, microCT analysis and hematoxylin and eosin staining showed that T2DM not only compromised bone microarchitecture but also reduced the BMD of the femoral metaphysis. This is possibly caused by more severe polyuria, metabolic imbalance of Ca and P, and accumulation of AGEs compared to typical T2DM. Taking our modeling approach of DOP, feeding with a high-sugar and high-fat feed and 25 mg/kg STZ injection, which artificially damages islet β cells, is different from the pathogenesis of T2DM.

Receptor activator of nuclear factor kappa-B ligand (RANKL) is predominantly secreted by osteoblasts and plays an indispensable role in osteoclast differentiation. Inhibition of the RANKL-RANK system is an important therapeutic target for the treatment of osteoporosis (35). OPG is a member of the tumor necrosis factor receptor superfamily of cytokines and has been shown to be closely related to the development of diabetes (36, 37). OPG binds to RANKL to prevent RANKL from binding to RANK, resulting in the differentiation of early osteoclasts into mature osteoclasts. In addition, OPG inhibits osteoclast differentiation and promotes osteoclast apoptosis (38). In diabetic condition, the accumulation of sugar contributes to the activation of multiple metabolic pathways such as the protein kinase C signaling pathway, the formation of glycation end-products and the polyol pathway which lead to the accumulation of AGEs, oxidative stress and the production of inflammatory cytokines (tumor necrosis factor alpha and interleukins 1, 6, and 17). Previous studies have demonstrated that soy isoflavones could exert osteoprotective effects *via* the RANKL/OPG ratio; however, most previous studies were based on post-menopausal osteoporosis instead of DOP (39, 40). RANKL and OPG are regulated by hormones (vitamin D and estrogen) and cytokines (TNFα and IL-1, IL-6, and IL-17) (41). Therefore, we believe that in bone tissue, accumulation of AGEs in osteoblasts and osteocytes, which leads to high-grade inflammation and low vitamin D status, decreases the ratio of OPG/RANKL expression. In our study, compared with the CON group, the DOP group showed significantly higher serum levels of RANKL and OPG, which were distinct from those in the bone tissue. Similar results were reported in other studies (42, 43). This may be due to the difference in the expression of OPG between bone tissue and other organs, such as the heart and liver (44). In the present study, we assessed the serum OPG and RANKL concentrations, as well as the mRNA and protein expression of OPG and RANKL in bone tissues and ROS17/2.8 cells. We found that after S-Equol treatment for 12 weeks, serum OPG was increased and serum RANKL was decreased in the treatment group compared with DOP rats. The mRNA and protein expression of OPG and RANKL in bone tissues and ROS17/2.8 cells had a significant change. The serum OPG/RANKL ratio, bone tissue and ROS17/2.8 cell OPG/RANKL mRNA expression ratio, and OPG/RANKL protein expression ratio were all increased in the S-Equol-treated rats compared with the DOP rats. These results indicated that S-Equol protects against T2DM-induced bone loss by upregulating the OPG/RANKL ratio.

Numerous studies have examined the effects of soy isoflavone and soy food intake on osteoporosis and osteoporotic fractures (45, 46). The main mechanisms include improvement of estrogen deficiency, promotion of osteoblast differentiation and proliferation (47, 48) and regulation of inflammatory cytokine levels in the bone marrow to inhibit osteoclast differentiation (47) and mineralization. However, most of the results have focused on post-menopausal osteoporosis. We observed that S-Equol increased bone density and altered bone microarchitecture in the DOP model, which has not been confirmed in previous experiments. A small number of experimental and cohort studies have documented that S-Equol can improve diabetes symptoms by regulating the gut microbiota and metabolites (49) and ameliorating insulin secretion failure (50, 51). Nevertheless, in the present study, S-Equol did not significantly improve the symptoms of diabetes. Taken together, we propose that the amelioration of osteoporotic bone architecture by S-Equol is primarily accomplished through osteoblast secretion of OPG and RANKL, independent of the improvement in glucose metabolism. During the experiment, it was observed that DOP induced ectopic adipocyte accumulation in the bone marrow cavities, and S-Equol effectively reversed this phenomenon. Type 2 diabetes, a chronic disease, affects osteoblast differentiation and is significantly mediated by the transcription factors PPARγ and Runx2 (52). Therefore, further studies are required to determine whether the differentiation of osteoclasts from bone marrow-derived mesenchymal stem cells could be affected by S-Equol.

The PI3K/AKT pathway has been shown to play an important role in the proliferation and differentiation of osteoblasts, regulating the ratio of OPG/RANKL (13, 53) and inhibiting osteoblast apoptosis (11, 54). Therefore, we presumed that S-Equol regulated the OPG/RANKL ratio *via* the PI3K/AKT pathway. We first examined the expression of pAKT/AKT in both bone tissue and osteoblasts using western blotting and qRT-PCR. We found that S-Equol increased the expression of pAKT and AKT. Akt phosphorylation was inhibited by Ly294002, and changes in the expression of OPG and pAKT/AKT were dramatically reversed. However, the expression of ERβ and RANKL did not have such an effect. After ERβ siRNA treatment, S-Equol did not increase the pAKT or OPG/RANKL ratio. This indicates that S-Equol first binds to ERβ and triggers the PI3K/Akt pathway activation. Finally, the OPG/RANKL ratio is regulated.

We acknowledge that there are limitations to our work. First, we could make no firm conclusions about what dose of drug is most effective in *in vivo* experiments. Secondly, in *in vitro* experiments, we failed to properly simulate cell state in all stages of osteoblast differentiation and in bone marrow cells, as a result, simulated results have some deviations from the *in vivo* experiment. Thirdly, We also failed to research the effects of S-Equol, which is the metabolite of the isoflavone daidzein by gut bacteria, on the gut microbiota.

## Conclusion

Our study confirms that S-Equol improves osteoporosis triggered by T2DM by promoting BMD and improving bone microstructure. Additionally, we targeted the regulatory mechanism by which S-Equol regulates the expression of OPG and RANKL to improve bone formation and prevent excessive bone turnover. Given the higher affinity of soy isoflavones binding to ERβ and natural compounds compared with anti-diabetic agents, the potential ability of S-Equol to prevent DOP should receive more attention.

## Transparency statement

The lead authors affirm that the manuscript is an honest, accurate, and transparent account of the study being reported; that no important aspects of the study have been omitted; and that any discrepancies from the study as planned have been explained.

## Data availability statement

The original contributions presented in this study are included in the article/supplementary materials, further inquiries can be directed to the corresponding authors.

## Ethics statement

The animal study was reviewed and approved by Experimental Animal Ethics Committee of the Third Military Medical University (AMUWEC2019350, 12 March 2019).

## Author contributions

SL, GZ, and HC performed some of the animal experiments. ZX performed the statistical analyses, conducted the molecular biology experiments, and drafted the manuscript. JX performed guidance and support in broadening our exposure to diabetes and osteoporosis research. XN and JW obtained funding, contributed to the experiment protocol, and critical revision of the manuscript for important intellectual content and approved the final version of the manuscript. All authors have read and approved the final manuscript.

## Footnotes

1
https://www.r-project.org

## Abbreviations

T2DM: type 2 diabetes mellitus
DOP: diabetic osteoporosis
OPG: osteoprotegerin
RANK: tumor necrosis factor receptor superfamily member 11B
RANKL: receptor activator of nuclear factor kappa-B ligand
ER α: estrogen receptor α
ERß: estrogen receptor ß
RANK: tumor necrosis factor receptor superfamily member 11A
OC: osteocalcin
PI3K: phosphatidylinositol 3′-kinase
AKT: protein kinase B
p-AKT: phosphorylated Akt
qRTPCR: quantitative reverse transcription polymerase chain reaction
siRNA: small interfering RNA
STZ: streptozotocin
ROS: reactive oxygen species
RT: room temperature.
